# Adenoid Cystic Carcinoma (ACC) of the Bartholin's gland misdiagnosed for three times: a case report

**DOI:** 10.1186/s12905-023-02320-4

**Published:** 2023-04-03

**Authors:** Moustafa Alhashemi, Sana Oubari, Aya Haji Mohamad, Mohamad Alhashemi, Obaida Kabel, Afaf Alhelue, Farah Fattal

**Affiliations:** 1 Faculty of Medicine, University of Aleppo, Aleppo, Syria; 2 Department of Gynecology and Obstetrics, Dar Altawleed Governmental Hospital, Aleppo, Syria; 3 Al-Tawleed Hospital, Aleppo, Syria

**Keywords:** Case Report, Adenoid Cystic Carcinoma, Misdiagnosed, Tumor, Neoplasm, Bartholin’s Gland, Recurrence

## Abstract

**Background:**

Adenoid cystic carcinoma (ACC) in Bartholin’s gland is an uncommon malignant tumor. These tumors have a vague clinical feature, so they are diagnosed late and discovered at a high-level stage. Our case presented Three Recurrences and Three times Misdiagnosis of Adenoid Cystic Carcinoma (ACC).

**Case presentation:**

We report a case of adenoid cystic carcinoma arising in Bartholin's gland of a 64-year-old female patient that appeared after three previous vulvar tumors were excised. The patient underwent bilateral radiotherapy which was performed on the perineum.

**Conclusion:**

ACC of the vulvar sweat glands is prone to misdiagnosis and delay in both diagnosis and treatment. As seen in our case, it was misdiagnosed three times as Chondroid Syringoma. Further studies need to be conducted to better understand the tumor prognosis, and its optimal treatment options.

## Background

Adenoid cystic carcinoma (ACC) in Bartholin’s gland is an uncommon malignant tumor. It is responsible for 2–7% of all carcinomas in the vulva and < 1% of all lower genital tract malignancies in females [1]. These tumors have a vague clinical feature and may confuse with other Bartholin’s lesions in differential diagnosis. As a result, they are diagnosed late and discovered at a high-level stage [2]. Perineural invasion, recurrence and metastasis can occur in different areas including lymph nodes, lungs, and bones. Patients have 59–100% survival rate for 10 years [3]. The ideal treatment of these neoplasms in the female genital tract remains unknown, some articles reported surgical operation and postoperative radiotherapy as a treatment [1, 4]. We present a rare case of recurrent Adenoid cystic carcinoma on the left labia minor of a 64-year-old woman. Which was 3 times misdiagnosed and treated as a chondroid syringoma.

## Case presentation

A 64-year-old female patient presented to our hospital with a vulvar mass. Over the previous 10 years, the patient noticed a slowly-growing mass in the left labia majora. Her vital signs were normal.

**Fig. 1:**
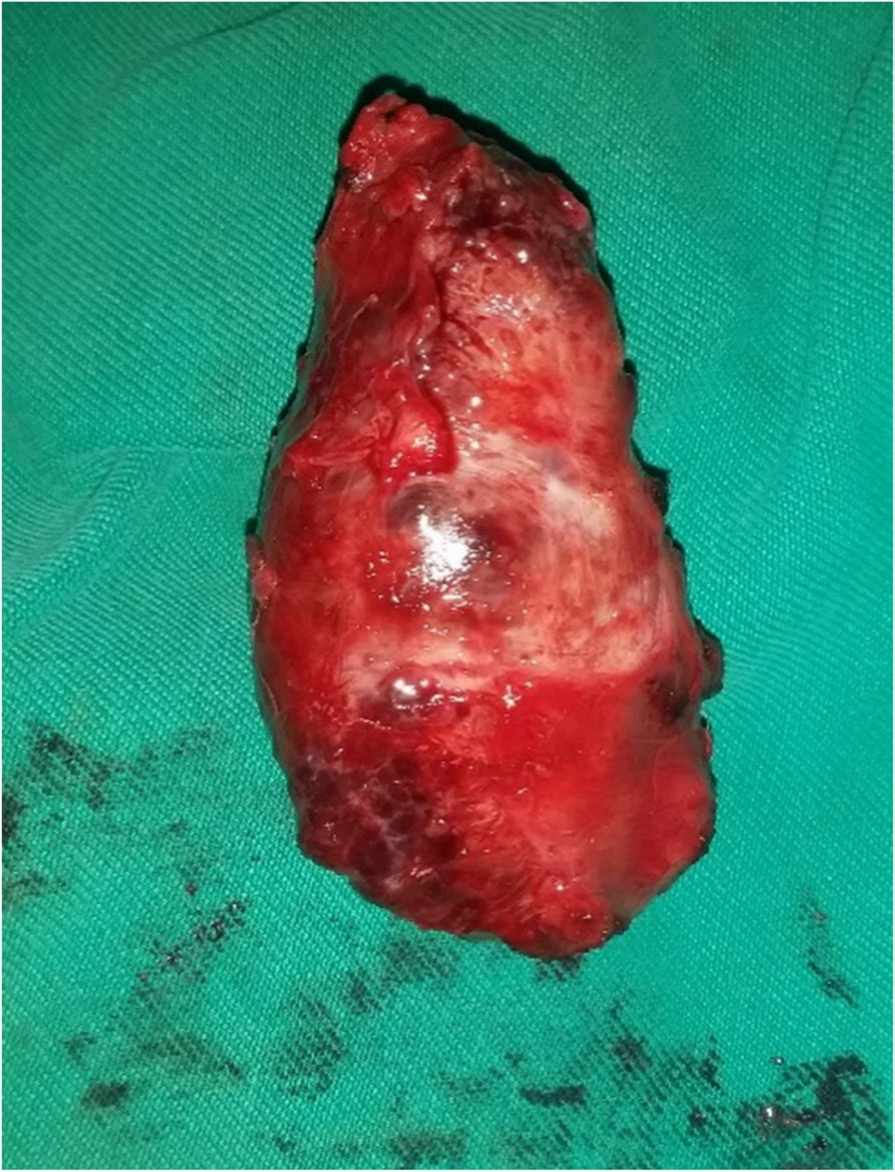
Macroscopy: vulvar tumor

Physical examination showed a firm, painless and immobile mass in the labia majora with an extension to the Mons pubis in size of 7 × 4cm. laboratory tests were normal. Surgical history showed that the patient had had a recurrence of vulvar tumors in the past. In 2000, The first mass was in Bartholin’s gland. It was excised in 2007, and the histopathologic examination revealed Chondroid Syringoma (mixed tumor of the skin). There was a proliferation of tubules and cystic structures lined by double-layered epithelium and filled sometimes with eosinophilic material and embedded in an abundant stroma made up of mucoid material and fibrous connective tissue sometimes hyalinised. After one year, a recurrence happened in the labia majora. The patient underwent a repeat excision, and the histologic examination revealed Clear Cell Hidradenoma, where it showed a lobular glandular proliferation of cuboid cells with no anaplasia. She also had benign breast lumpectomy and Cholecystectomy. Her medical history was unremarkable. Transvaginal Ultrasound revealed a heterogeneous, regular, hypervascular mass that is located under the skin directly. The Ultrasound’s mass dimensions were 4.2 $\times$ 8 cm. The abdominal and pelvic multi-slice CT scan revealed a 3.8 × 7.2 cm heterogeneous, well contrast-enhancing, high-density area located on the right side of the left labia majora with an extension to the anterior border of the perineum. CT scan also showed a low-density focus in the left hepatic lobe that is suspected to be a simple cyst or Haemangioma. The mass was excised and sent to the Pathology department. Grossly, it was a 7 × 3 × 4 cm, grey-coloured tumor (Fig. 1) and the conclusion of the histopathologic examination was Chondroid Syringoma. After 3 months of follow-up, the patient had another recurrence in the Left Labia minora close to the fourchette. It was excised, and the histopathologic examination revealed Adenoid Cystic Carcinoma.

Histopathology showed proliferation of uniform small cells having dark, compact nuclei and scant cytoplasm, reveals some mitotic figures.

**Fig. 2:**
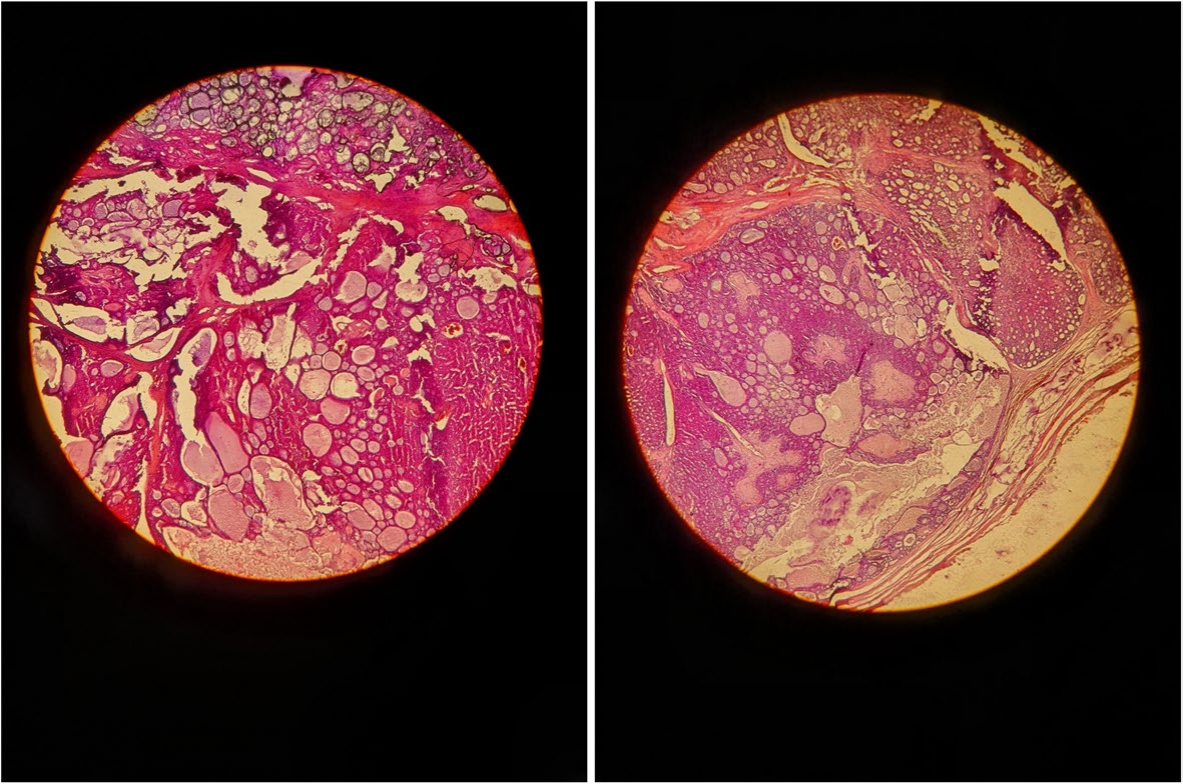
Microscopy: epithelial and stromal components. Epithelial components made up of elongated branching tubular structures with two layers of cells. The stromal components are made up of chondroid to fibrous/hyaline stroma

There are epithelial components that made up of elongated branching tubular structures with two cell layers. The stromal components are made up of chondroid to fibrous/hyaline stroma (Fig. 2). the patient underwent 25 sessions of bilateral radiotherapy, one session per day, 5 days a week, for a month and a half. The radiotherapy was performed on the perineum. A new CT scan was ordered after the radiotherapy, and the results revealed no metastases. Due to the repeated recurrence, it is believed that the first three recurrences were misdiagnosed and were malignant tumors instead of benign ones. Unfortunately, immunohistochemistry could not be performed due to sanctions and lack of resources in the case's country.

## Discussion and conclusion

Bartholin gland (BG) is a pea-sized impalpable gland, located bilaterally at 4 and 8 o’clock in the base of the labia minora. It lubricates the vagina during sexual arousal or intercourse together with other glands, because of that, removing the BG does not affect lubrication [5].

Many lesions affect the BG, including benign tumors and tumor-like lesions, premalignant lesions and malignancies, mesenchymal lesions, lymphoma and inflammatory lesions with cysts and abscesses being the most common.

Bartholin's gland malignancies include many histological subtypes; such as: adenocarcinoma, squamous cell carcinoma, transitional cell carcinoma, adenoid cystic carcinoma, and undifferentiated adenocarcinoma. Adenocarcinoma and squamous cell carcinoma are the most common malignancies in Bartholin's gland, while ACC is considered to be a rare genital malignancy [2].

ACC is a rare tumor, common in the head and neck area, mainly in the minor salivary glands, the parotid and sublingual salivary glands, glandular tissue of the nasal passages and tracheobronchial tree [3]. ACC in Bartholin’s gland is unique, as it only represents 10–15% of BG malignancies, 2–7% of the carcinomas in the vulva, and less than 1% of the genital malignancies. The median age is 47 [1].

The diagnostic criteria for ACC were re-established in 1972 to better meet with the clinical findings of the tumor as follows: 1) Histological findings for the transitioning areas from normal to neoplasia. 2) The tumor must be originating from Bartholin's gland. 3) No evidence of a previous primary or concurrent primary tumor anywhere else [4].

ACC is a slow growing tumor, which invades the perineural region and lymphatic vessels, and reoccurs locally, but metastasizes distantly and usually to the lungs [1]. Our patient did not have hematogenous or lymphatic metastasis, which is uncommon.

Symptoms are usually non-specific and include bleeding, pruritus, pain, local inflammation, dyspareunia, and a painless lump at the site of the BG. ACC infiltrates the perineural spaces causing local itching and burning sensations [4].

These vague symptoms made it difficult to differentiate ACC from cysts and other benign or malignant tumors. As seen in our case, our patient suffered only from a painless lump, and the lesion was encapsulated, which mimics the symptoms of benign lesions [2]. The cyst was excluded, because it is more common in young women, and causes symptoms of pain during walking, sitting, and intercourse [5], which were absent in this case. After excluding the cyst, and considering the patient’s age and the vague presentation as a painless tumor with no metastasis, the patient was 3 times misdiagnosed with Chondroid Syringoma (CS). CS – which is also known as pleomorphic adenoma or benign mixed tumor- is a rare encapsulated, slow-growing, well-defined, painless and solid tumor [6]. After the third recurrence, the biopsy confirmed the diagnosis of Bartholin's gland ACC.

The histology of Bartholin's gland ACC is similar to the salivary gland ACC. The cribriform pattern is the most typical characterization, which is formed by nests of tumor cells interrupted by basophilic basement membrane materials [3, 4].

Due to the rarity of this tumor, there is lack of studies regarding the optimal treatment. Depending on how deep the tumor has invaded, there are two surgical approaches; simple excision, and radical vulvectomy, with or without inguinal and/or femoral lymph nodes excision [4].

Yet, there has been a debate whether the surgical removal with clear resection margins is the most important aspect of treatment to prevent recurrences. Yang et al. literature review showed that negative resection margins may not be as strong evident as previous for predicting local recurrence. Positive resection margins rate was 48% after simple excision, and 30% after radical vulvectomy. However, when comparing recurrence rates, it was 52.9% in patients with positive margins, in comparison with 52.1% in patients with negative margins. Although, half of the patients with positive margins received radiotherapy as well [1].

In our case, the tumor was located in the labia majora, and was encapsulated. Due to the misdiagnosis, only the encapsulated tumor was resected in the previous surgeries with negative resection margins. However, she had three recurrences in the past 22 years.

Adjuvant radiotherapy is highly recommended in cases of positive resection margins or perineural infiltration [1]. Rosenberg et al. study proved that external beam radiation has a remarkable local control of the tumor in patients with positive resection margins [4]. The recurrence rate was higher in patients not receiving radiotherapy. This highlights the possible role of radiotherapy in preventing local recurrences [1]. In our case, and considering the past three recurrences, radiotherapy was recommended. After the final diagnosis of Bartholin's gland ACC, the patient underwent 25 sessions of bilateral radiotherapy.

While chemotherapy has proven to be effective in cases of ACC of the salivary gland, there is still a little evidence regarding its role in Bartholin's gland ACC [1].

Metastasis to the ipsilateral inguinal-femoral lymph nodes is rare, occurring in only 10% of all cases. While metastasis to the contralateral lymph nodes was not reported before. However, there is no reported indication regarding lymphadectomy, except when there is suspicion of lymph nodes invasion [4]. Our patient did not have lymph node dissection because the regional lymph nodes were not involved.

From above we conclude that radical vulvectomy and radiation are recommended in cases of ACC with local invasion or recurrences. Table 1 shows a comparison to differentiate ACC from Syringoma from clinical gross histomorphological features.

**Table 1:** A comparison to differentiate ACC from syringoma from clinical and gross histomorphological features

	**Chondroid Syringoma**	**Adenoid Cystic Carcinoma**
Clinical Manifestation	Painless, solid, ovoid or round mass	A painless lump with unspecific symptoms including local inflammation, pain, bleeding, pruritus and or dyspareunia with perineural infiltrations [4].
Histological features	Epithelial components include cuboidal, basaloid, squamous, spindle cell, plasmacytoid, and clear cells usually forming sheets or ducts Myoepithelial components contain spindle-shaped or plasmacytoid cells forming sheets, or appear as a reticular pattern Mesenchymal components are myxoid/mucoid, cartilaginous or hyalinised [6].	Acellular spaces containing mucin and hyalinized material Uniform small cells forming cords and nests surrounding the acellular spaces The tumor cells are basaloid, with little cytoplasm and regular nuclear structure [4].

In conclusion, ACC of the vulvar sweat glands is prone to misdiagnosis and delay in both diagnosis and treatment, due to its non-specific characterization and slow growth. As seen in our case, it was misdiagnosed three times as Chondroid Syringoma. However, although ACC is uncommon, it should be considered in any slow growing, recurrent lesion with no response to conventional treatment. Local control of the tumor is recommended, as it has high tendency rates for local recurrence. Further studies need to be conducted to better understand the tumor prognosis, and its optimal treatment options, as there are no clear treatment guidelines for ACC of the Bartholin gland.

## Data Availability

All data generated or analysed during this study are included in this published article and its supplementary information files.

## Abbreviations

ACC: Adenoid Cystic Carcinoma
BG: Bartholin gland
CT: Computed Tomography
